# New Methodology to Produce Sets of Valence Bond Structures with Enhanced Chemical Insights

**DOI:** 10.1021/acs.jctc.2c01000

**Published:** 2023-05-15

**Authors:** Sourav Roy, Avital Shurki

**Affiliations:** Institute for Drug Research, School of Pharmacy, Ein Kerem Campus, The Hebrew University of Jerusalem, Jerusalem 9112001, Israel

## Abstract

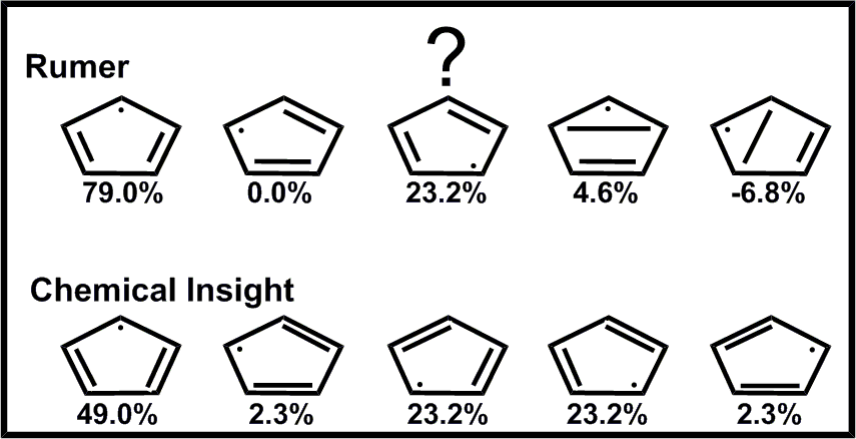

The valence bond (VB) theory uses localized orbitals, and its wave function is composed of a linear combination of various VB structures which are based on sets of spin functions. The VB structures are not unique, and different sets are used, Rumer sets being the most common for classical VB due to their advantage as being both easily obtained as linearly independent and meaningful. Yet, Rumer rules, which are responsible for the simplified process of obtaining the Rumer sets, are very restrictive. Furthermore, Rumer sets are best suited for cyclic systems; however, in noncyclic systems, structures resulting from Rumer rules are often not the most intuitive/suitable structures for these systems. We have developed a method to obtain chemically insightful structures, which is based on concepts of chemical bonding. The method provides sets of VB structures with improved chemical insight, which can also be controlled. Parallel to the Rumer structures, the chemical insight sets of structures are based on electron pair coupling, and hence, pictorially can be drawn similarly to the Lewis structures. Yet, different from Rumer rules, the chemical insight method, being more flexible, allows larger combinations of bonds as well as larger combinations of structures in the sets it offers, resulting in many more possible sets that are better adapted to the systems studied.

## Introduction

Classical valence bond (VB) theory was first formulated in 1927 by Heitler and London,^1^ to describe the bond in the hydrogen molecule as a spin-pair. It was then further developed to describe n-electron systems using the Heitler-London-Slater-Pauling (HLSP) functions.^2−5^ As an extension of the original Heitler-London approach, these HLSP functions are constructed by coupling the spins of pairs of electrons to form singlets, leaving the remaining 2*S* electrons (where *S* is the overall system’s spin) unpaired. The VB wave function is then composed of a linear combination of these spin functions, where each is an eigenfunction of *S*^2^ and *S*_*z*_. There are many different ways to couple pairs of electrons, resulting in an overcomplete set of functions. Thus, Rumer designed some useful graphical rules to select a complete linearly independent set of spin functions that span the subspace of the particular system addressed.^4^ Based on the Rumer scheme, active single electron orbitals are placed in an imaginary circle and the electrons are spin paired to generate all possible VB structures following one condition, namely not displaying crossing bonds. These rules were then extended to nonsinglet states by Simonetta et al.^6^

Other methods have been developed to construct an independent set of spin functions within the VB approach. These include, for example, the genealogical basis, also known as the Yamanouchi-Kotani basis.^7−9^ Here the spin functions are constructed by coupling individual electron spins, one at a time, using the usual rules for combining angular momenta. This successive coupling is done so that each partial spin function is an eigenfunction of *S*^2^.^9−11^ The graphical representation of this construction path is called the branching diagram.^9−11^ Another example involves the Serber basis set, which is obtained by coupling the spins of pairs of electrons to form either singlets or triplets. These pairs are then coupled together sequentially, in the same way as for the genealogical functions, to give the overall spin, *S*.^9,12,13^

The latter methods (including the genealogical, Yamanouchi-Kotani, and Serber) involve a recursive process but result in a complete set of orthonormal spin functions, which are often used by, e.g., the spin-coupled generalized valence bond (SCGVB) approach.^14,15^ The Rumer spin functions, on the other hand, are not orthogonal, but simpler to obtain. Furthermore, the interpretation of the Rumer functions to chemically intuitive structures, such as Lewis structures, including both covalent and ionic structures, is straightforward. This is somewhat less straightforward for the other two methods. Hence, classical VB commonly utilizes Rumer structures.^16−19^

Rumer set was originally constructed for cyclic systems with one active orbital per atom, in which case the set also follows the symmetry of the system (for singlet states) and contains maximum number of perfectly paired structures. As such, it is usually chemically meaningful for these systems. However, when the system is not cyclic, contains an odd number of electrons, and/or contains more than one active electron per atom, then a symmetric set cannot be guaranteed. Furthermore, the structures obtained may not contain all perfectly paired structures of interest, and some chemically meaningful structures may be missing (see, e.g., structure 6 in Scheme 2c). Finally, in some cases, the Rumer set may result in nonphysical structures or structures which are not physically preferred (e.g., singlet paired electrons on the same atom, see structure 9 in Scheme 5 and related text).

Some of these problems can be solved by rearranging the orbitals, but the number of rearrangements or permutations of orbitals can be very large (e.g., for a system with eight active orbitals there are 8! (= 40320) possible ways to number the orbitals), and the search for a set with proper structures, therefore, may become very tedious. In other cases (e.g., odd number or electrons), Rumer structures cannot provide all expected important structures, due to the strictness of the rules.

The structure set is not unique and there are several ways to choose different independent VB sets. It is useful, therefore, to be able to choose a meaningful set. We introduce here a new methodology for choosing sets of structures with chemical insight which are adapted for any system without the restrictions of the Rumer rules. We also show that our chemical insight methodology can reduce and often overcome the limitations of Rumer sets. The sets can be used for any VB calculation including both classical VB as well as the SCGVB.

## Methodology

The strategy involves generation of all possible HLSP functions for a given system. The structures are then ranked using several criteria based on chemical intuition and possibly also user’s preferences, as described below. The different ranks are gathered into a unified ranking. Finally, a set of linearly independent and highly ranked structures is chosen, resulting in a set of structures with chemical insight.

### Structure Generation

For a system with *n* active orbitals and spin *S* we define *N* as the number of singly occupied orbitals. The remaining *n* – *N* orbitals will either be vacant (for positively charged species) or doubly occupied (the lone pairs of neutral or negatively charged species). Hence, the total possible number of HLSP structures is given by (see SI for a detailed explanation):
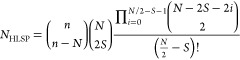
1

A list of all possible () singlet-coupled pairs is generated. Each structure should have (*N*/2 – *S*) singlet-coupled pairs. Thus, groups of (*N*/2 – *S*) pairs are chosen (in each group any orbital is involved in only one bond); lone pairs (or vacant orbitals) are assigned for each group (placed in orbitals that were not involved in any of the pairs within the group) and the structure is supplemented by unpaired electrons (again on orbitals that were not involved in either singlet-coupled pairs or lone pairs/vacant orbitals).

### Diverse Structure Ranking

**Scheme 1:**
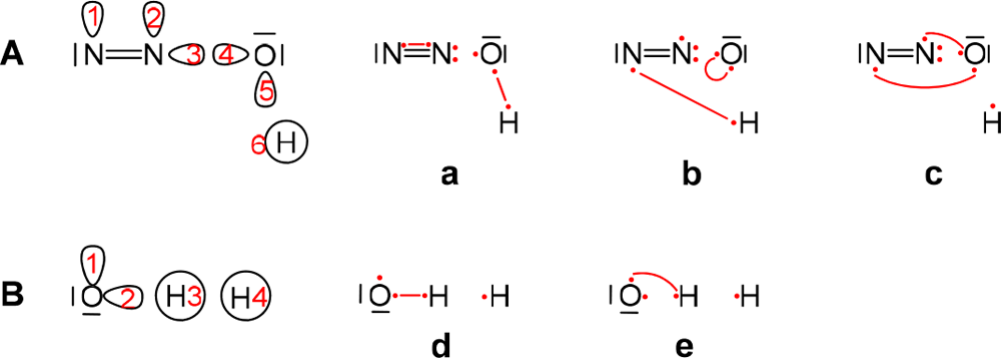
Numbering of the Active Orbitals of the Systems (A) N_2_O + H → N_2_ + OH with 7e/6o along with Three Possible Structures (**a**–**c**) and (B) OH + H → O + H_2_ with 4e/4o along with Two Possible Structures (**d**, **e**) Active electrons appear as dots with the lines connecting them indicating covalent bonds.

A number of criteria can be used to rank the various structures. We used five different criteria which are described below along with the corresponding scoring procedure that was used to rank the structures. Note that the highest quality structures, based on the different criteria, are ranked 1 and the values increase as the quality of the structures decreases:

1. Predefined bonds: Bonds the user considers as important to have in the final set of structures (e.g., bonds between orbitals 1–2 and 5–6 in structure **a** (Scheme 1A) or bond 3–4 which is not available in Scheme 1A). The score for each structure, in this case, is given by
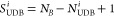
2
where *N*_*B*_ is the overall number of bonds in each structure and *N*_UDB_^*i*^ is the number of user-defined bonds (UDB) available in structure *i*. This criterion is useful particularly for describing reactions, as it can enhance the appearance of the bonds which are formed/broken.
2. Predefined radicals: Radicals the user considers as important to have in the final set of structures (e.g., radical on the oxygen (orbital 4) as in structure **a** (Scheme 1A) or radical on the hydrogen (orbital 6) as in structure **c** (Scheme 1A). The score of each structure, in this case, is given by
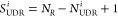
3
where *N*_*R*_ is the overall number of singly occupied orbitals, occupied by unpaired electrons in each structure and *N*_UDR_^*i*^ is the number of user’s defined singly occupied orbitals, occupied by unpaired electrons (referred to as user-defined radicals (UDR)), available in structure *i*.
3. Inter versus intra-atomic bonds: Interatomic bonds (e.g., bonds between orbitals 1–2 or 5–6 in structure **a** (Scheme 1A)) are preferred over intra-atomic bonds (e.g., the bond 4–5 in structure **b** (Scheme 1A)). The score for each structure, in this case, is given by
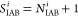
4
where *N*_IAB_^*i*^ is the number of intra-atomic bonds (IAB) available in structure *i*.
4. Bond length: Electron pairing is favored; the closer the atoms (e.g., bonds between orbitals 1–2 or 5–6 in structure **a** (Scheme 1A) are preferred over the bond 1–6 in structure **b** or 1–5 in structure **c** (Scheme 1A)). In order to score each structure in this case, the relative distance *D*_NAB_^*i*^ between the atoms in the geometry of interest and in a hypothetical geometry in which all the structure bond lengths are “ideal”, is estimated by
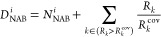
5
*N*_NAB_^*i*^ is the number of neighboring atom bonds (NAB) in structure *i*, namely, bonds with *R*_*k*_(*AB*) ≤ *R*_*k*_^*cov*^(*AB*), where *R*_*k*_(*AB*) is the actual bond distance between atoms A and B (in the given geometry) and *R*_*k*_^*cov*^(*AB*) is the sum of the covalent radii of atoms A and B deduced from crystallographic data.^20^ The structures are then sorted from lowest to highest based on *D*_NAB_^*i*^. The sequential number of each structure in the resulting sorted list serves as the structure score, *S*_NAB_^*i*^. We note that this criterion can lead to different structure scoring at different geometries. It is, thus, recommended not to have bond length as the only criterion or as the criterion with the highest priority, particularly for the description of reactions.
5. Orbital symmetry: Electron pairing is favored between orbitals of the same symmetry rather than different symmetries (e.g., the bond between orbitals 2–3 in structure **d** (Scheme 1B) is preferred over the bond 1–3 in structure **e** (Scheme 1B)). The score given to each structure in this case is given by
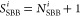
6
where *N*_SBB_^*i*^ is the number of bonds between orbitals of different symmetries in structure *i*.

We note in this respect that formal charges were not considered because structures with different formal charge distribution belong to different spaces and cannot be compared. Hence, sorting is done separately for each distribution of doubly occupied/vacant orbitals.

### Unified Structure Ranking

The five different criteria result in five different quality scores. However, a highly ranked structure based on one quality score does not necessarily guarantee a high rank based on the other quality scores. Hence, the different quality scores are merged sequentially according to a preset order of priorities, and a single score is eventually assigned to each structure, that is, the score of two different criteria is merged into one. The resulting score is then merged with the score of the next criterion according to a preset order of priorities and the process continues until all scores are merged. This is done by following eq 7:
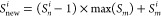
7where *S*_*n*_^*i*^ and *S*_*m*_^*i*^ are the scores for structure *i* based on criteria *n* and *m* (*n* being the criterion with the higher priority), max(*S*_*m*_) is the maximum score for criterion *m*, and *S*_new_^*i*^ is the resulting merged score. This *S*_new_^*i*^ then serves as the new *S*_*n*_^*i*^ and is merged with the next criterion score.

The resulting unified score given to each structure keeps the order of priority of the different criteria as well as the sequence of structures within the individual criteria. This unified score is referred to as the quality score, as it indicates the quality of the structures based on the different criteria.

### Selection of an Independent Structure Set

A system with *N* singly occupied orbitals (i.e., *N* electrons in *N* orbitals) involves *N*_*IS*_ linearly independent structures:^9,21^
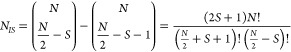
8

If the system has lone-pairs or vacant orbitals, this number should be multiplied by  which is the number of different ways to place (*n* – *N*) lone pairs or vacant orbitals in overall *n* orbitals. However, structures of any given distribution of lone-pairs/vacant orbitals form linearly independent subspaces. Thus, selection of an independent set is carried out for each subspace separately.

The structures are sorted based on the unified scoring. Selection of a complete independent set starts with the first two structures from the sorted list, which are the structures with the highest score. Linear dependency of the next structure from the sorted list with the chosen set is then examined, and if found independent, it is added to the set. This examination is done by use of the Gramian matrix, *G*, which is an overlap matrix given by
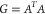
9where *A* is the matrix of vectors assigned to the structures (written as columns) and *G*_*ij*_ is thus, an inner-product of the structure vectors *i* and *j*.^22^ Invertibility of *G* (i.e., det *G* ≠ 0) indicates that the structures are linearly independent.^22^

Hence, each structure which is a linear combination of several spin–orbital determinants is expressed as a vector using the coefficients of these determinants in the structure’s expansion. The Gramian matrix of the selected set with the new structure, the linear dependency of which is examined, is produced, and its determinant calculated. If found to be zero, the structure is rejected and the linear dependency of the next structure in the list is examined. The process is repeated until *N*_*IS*_ independent structures are selected.

### Computational Details

Ab initio VB calculations were performed with the XMVB program, version 3.0;^23−25^ MO-based calculations were performed with *GAMESS US*, version R1.^26^ Four different test cases were calculated using different VB sets: C_2_, C_5_H_5_ in the *C*_2*v*_ symmetry, and the transition states of the following two reactions: H + OH → O + H_2_ and HNC → NCH. Geometries for C_5_H_5_ and C_2_ are based on the QCISD/6-31++G(d,p) level. Geometries for the two transition states are based on the QCISD/MG3 level with the spin-restricted formalism for closed-shell systems and the fully spin-unrestricted formalism for open-shell systems as reported earlier.^27^ Geometries can all be found in the Supporting Information.

All VBSCF calculations utilized Pople-type basis set, 6-31++G(d,p)^28,29^ using the Cartesian (6D) orbital representation. Core orbitals were allowed to optimize for the C_5_H_5_ and C_2_ systems and were frozen at the spin-restricted Hartree–Fock level for the transition states of the two reactions. Valence electrons were treated as described below for each system. For clarity, the description is based on the symmetries of the corresponding MOs at the calculated geometry.

**Scheme 2:**
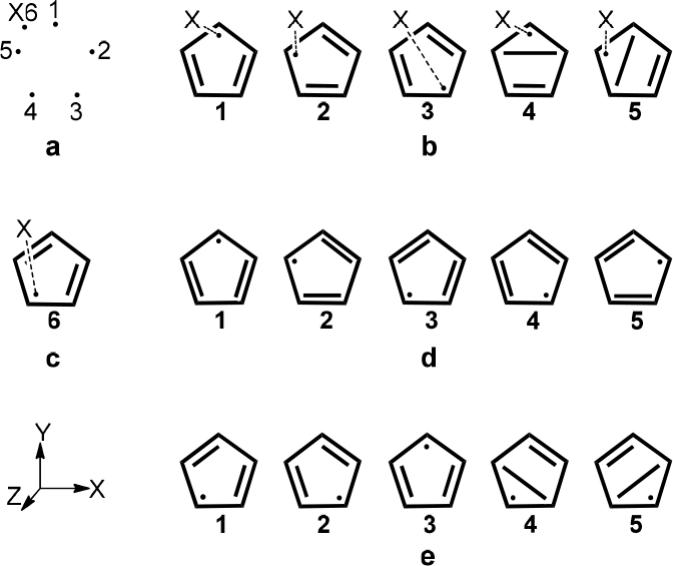
Application of Rumer’s Pictorial Method to the π Electrons of C_5_H_5_ System in Its Doublet State (see text for details) Using 5*e*/5o (a) Numbering of the orbitals (b) allowed structures based on Rumer rules using the orbital numbering in 2a. Dashed lines between the dummy center (X) and any of the orbital centers are eliminated in the final structures. (c) Example of a forbidden structure, **6**_**,**_ based on Rumer rules. (d) An ideal complete set of Kekulé structures for π electrons of the doublet state of C_5_H_5_. (e) Additional Rumer set using a different orbital ordering along with the system’s axes (for orbital numbering see (a). The σ skeleton is always drawn for all the structures in sets b–e.

C_5_H_5_: This system is a doublet. It is described by five active electrons in five active orbitals, 5e/5o, resulting in five VB structures for a complete covalent set (Scheme 2). The five active orbitals are π type, one on each carbon. In addition, there are ten inactive valence orbitals occupied by 20 electrons; all are σ type delocalized over the whole C_5_H_5_ system.

TS of H + OH → O + H_2_: This system is a triplet. It is described by four active electrons in four active orbitals, 4e/4o, resulting in three VB structures for a complete covalent set (Scheme 5). Three active orbitals are σ type, one on each fragment (H, H, and O), and one is π type on the oxygen. In addition, there are two inactive valence orbitals occupied by four electrons; both are lone pairs of oxygen (one σ and one π).

TS of HNC → NCH: This system is a singlet. It is described by six active electrons in five active orbitals, 6e/5o, resulting in ten VB structures for a complete covalent set (a subset of these structures is shown in Scheme 6). Each of the N and C fragments has two active orbitals, one parallel to the CN bond, pointing to the opposite direction, and one perpendicular to that bond and H has one active orbital. In addition, there are two inactive valence orbitals occupied by four electrons; delocalized over the two C and N fragments, both describing the CN bond (one σ and one π).

C_2_: This system is a singlet. It is described by eight active valence electrons in eight active orbitals, 8e/8o, resulting in 14 VB structures for a complete covalent set (Schemes 7 and 8). Each C atom has two σ type active orbitals and two π type active orbitals.

The weights of the VB structures within each set are based on the Coulson-Chirgwin formula:^30^
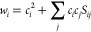
10where *c*_*i*_ are the coefficients of the *i*^*th*^ VB structure and *S*_*ij*_ is the overlap between the *i*^*th*^ and *j*^*th*^ VB structures.

To dictate whether the Gramian matrix is invertible, the matrix is diagonalized, and all the absolute values of the eigenvalues are required to be higher than a threshold value of 1 × 10^–11^.

## Results and Discussion

### Missing Structures

#### Cyclic Systems with Odd Numbers of Electrons

The restrictions of the Rumer rules on the structures lead to forbidden structures. However, sometimes these forbidden structures may be important. Cyclic systems with *N* electrons in *N* orbitals, where *N* is an odd number, can serve as an example for such a case.

According to the Rumer rules, as mentioned earlier, structures are obtained by arranging the orbitals in a circle and spin-pairing them in all possible ways while eliminating structures where bonds are crossing.^4^ When the number of electrons is odd (e.g., C_5_H_5_), a “dummy” center is introduced for each unpaired electron in the system in sequential order (X6 in Scheme 2a), and the procedure described above is repeated while considering the dummy centers as if they are extra electrons, with the exception that bonds between these centers cannot be formed. Hence, bonds between any unpaired electron and these dummy centers must not cross an actual bond.^6^Scheme 2b shows a resulting set of allowed Rumer structures (**1**–**5**). Finally, the dummy centers are eliminated from the resulting structures, and electrons occupying orbitals which were bonded to these dummy centers represent the unpaired electrons of the structures.

Thus, for systems with singly occupied orbitals, Rumer rules allow pairing only between orbitals the numerical difference of which is odd, as otherwise crossing of the bonds cannot be avoided. As a result, in odd electron cyclic systems, the bond between the first and last orbitals, for example, cannot appear and structures involving that bond, are forbidden (e.g., Scheme 2c). Ideally, a proper description of the system in these cases should involve N Kekulé type structures, each assigning an unpaired electron to a different center and spin pairing the remaining bonds on neighboring carbons (see, e.g., Scheme 2d for the π electrons of C_5_H_5_). Yet, at least two of these structures (structures **3** and **5** in the C_5_H_5_ example, Scheme 2d) cannot be included in a Rumer set of structures due to its inability to describe a bond between the first and last orbitals (orbitals 1 and 5 in Scheme 2a).

Permutation of the orbitals may turn bonds which are forbidden in the original numbering scheme to allowed bonds, suggesting that maybe the problem of the missing structures described above can be solved. However, while in some cases this indeed solves the problem, in this case, for example, it cannot. To understand why, we must deepen our understanding of the number of allowed bonds in any Rumer set.

#### Overall Number of Different Allowed Bonds in a Complete Set of Structures

The overall number of different bonds a Rumer set describes, *n*_*b*_^set^, is limited due to the restrictions of the Rumer rules and is given by eq 11 (see Supporting Information for more details):
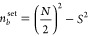
11

Using the proposed (chemical insight) method, all bonds are allowed with no limitation on the number, as long as the set spans the space, and each set can include up to  bonds (see Supporting Information for details).

For the π electron system of C_5_H_5_ in its doublet state, a complete set of structures includes five structures. Eq 11 predicts that any Rumer set for that system involves six different bonds. However, the five Kekulé structures set, which forms a complete set, involves only five different bonds, suggesting that when Rumer rules are applied that set cannot be obtained regardless of the orbital numbering. The proposed (chemical insight) method, on the other hand, gives the set of five Kekulé structures as the highest quality set.

#### Symmetry

The Rumer set shown in Scheme 2b is not intuitive, as it does not enable description of the molecule’s symmetry in a *D*_5*h*_ geometry. In this particular system, symmetry breaking was demonstrated in numerous strudies.^31−33^ Thus, a wave function that follows *D*_5*h*_ symmetry is not expected even with the five Kekulé structures. Therefore, we will focus on the *C*_2*v*_ geometry of C_5_H_5_, with the *Y*-axis depicted in Scheme 2e serving as the principal symmetry axis.

The Rumer set shown in Scheme 2b does not follow rotation about this *Y* axis and, thus, does not follow the corresponding *C*_2*v*_ symmetry. Permutation of the orbitals, as mentioned earlier (see, e.g., ref (34)), results in new sets, some of which may follow the *C*_2*v*_ symmetry. Scheme 2e displays the most intuitive Rumer set that follows this *C*_2*v*_ symmetry, obtained by reordering the orbitals (other possible Rumer sets that follow the symmetry involve crossing of the bonds and are therefore less intuitive; see Scheme S2 in Supporting Information). Structures **4** and **5** in Scheme 2e involve a long bond which is less favorable than the bonds described in the Kekulé set (Scheme 2d). Furthermore, Table 1 summarizes VBSCF Coulson-Chirguin weights obtained for the five Kekulé structures set (Scheme 2d), and the two different Rumer sets depicted in Scheme 2b,e for the covalent state of the π-electrons of C_5_H_5_ in *C*_2*v*_ geometry. The weights for set 2b are different for the different structures and do not reflect any symmetry while those of set 2e follow the system’s symmetry. However, the weights of both these Rumer sets include unphysical non-negligible negative values for some of the structures, suggesting there is some problem with the presentation of the wavefuction. The weights of the Kekulé set, on the other hand, are all positive, indicating that the resulting interpretation of the wave function is balanced. These observations suggest that the Kekulé set is better suited not only for the description of the molecule in the highest (*D*_5*h*_) symmetry but also in the *C*_2*v*_ geometry.

**Table 1:** VBSCF Weights (in %) of the Five VB Structures within the Kekulé Set (Scheme 2d) and Two Different Rumer Sets (Scheme 2b,e) Describing the π-Electrons of C_5_H_5_ in *C*_2*v*_ Symmetry

	Kekulé	Rumer Sets	
Structure	Set 2d	Set 2b	Set 2e
1	49.0	79.0	29.5
2	2.3	0.0	29.5
3	23.2	23.2	48.9
4	23.2	4.6	–3.9
5	2.3	–6.8	–3.9

### The Difficulty to Obtain Certain Structures in Noncyclic Systems

**Scheme 3:**
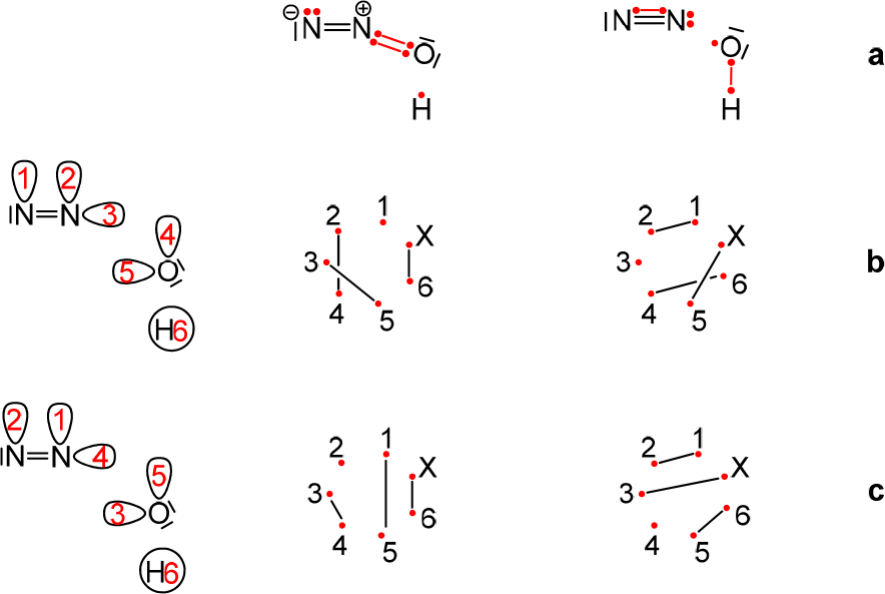
Influence of Orbital Ordering on the Quality of the Rumer Sets for the N_2_O + H• → N_2_ + •OH Reaction Using 7*e*/6o (a) Chemically meaningful structures. Active electrons appear as dots with lines connecting them indicating covalent bonds. (b, c) The simple orbital ordering and a careful choice of orbital ordering, respectively, along with a description of the structures depicted in (a) using the Rumer scheme, showing these structures are forbidden in (b) and allowed in (c) based on Rumer rules, using the corresponding orbital numbering.

**Scheme 4:**
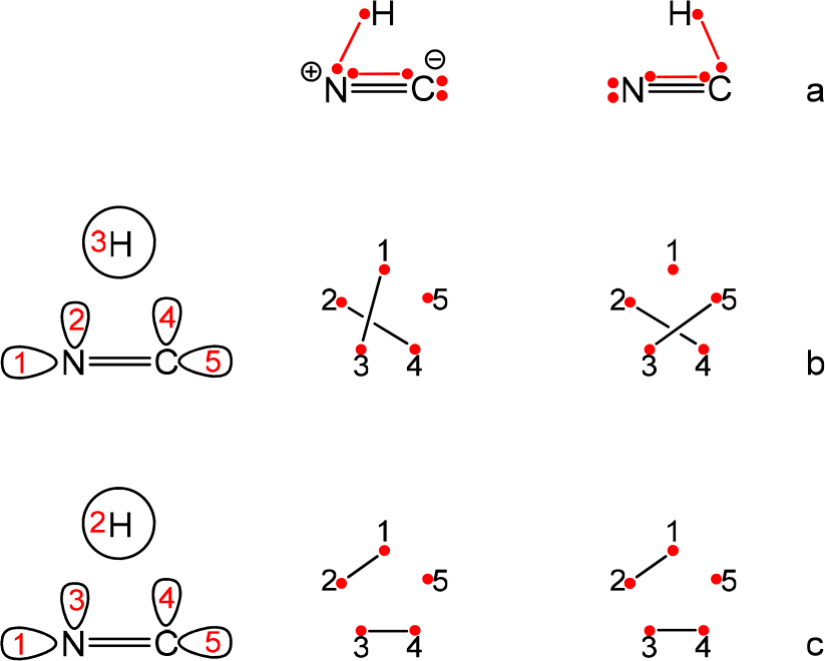
Influence of Orbital Ordering on the Quality of the Rumer Sets for the HNC → NCH Reaction Using 6*e*/5o (a) Chemically meaningful structures. Active electrons appear as dots with lines connecting them indicating covalent bonds. (b, c) The simple orbital ordering and a careful choice of orbital ordering, respectively, along with a description of the structures depicted in (a) using the Rumer scheme, showing these structures are forbidden in (b) and allowed in (c) based on Rumer rules using the corresponding orbital numbering.

The Rumer sets are well suited for cyclic systems with an even number of electrons. However, for noncyclic molecules, Rumer structures do not guarantee the best set, in terms of chemical insight, and some meaningful structures may be absent. Scheme 3a shows two structures a chemist would consider as important for the description of the reaction N_2_O + H• → N_2_ + •OH. These structures are forbidden using the most popular orbital arrangement an inexperienced user would probably choose (Scheme 3b). Similarly, Scheme 4a lists two highly important structures for the description of the reaction HNC → NCH which, again, are forbidden using the most common assignment of orbital numbering (Scheme 4b). We should clarify here that the problem in both these cases is not manually choosing these two structures but rather finding a full Rumer set that includes these two structures.

As mentioned earlier, a well-chosen permutation of these orbitals may remove the restriction of these structures (Schemes 3c and 4c). However, the number of different possible permutations, which result in different possible Rumer sets, *n*_sets_^Rumer^ is
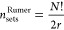
12where *r* is the rotation order of the principal axis of the relevant Rumer cycle for even *N*, and *r* = 1 for odd *N*. This number should also be multiplied by  for systems that involve (*n* – *N*) lone pairs or vacant orbitals. Depending on the system, this can be a large number and the process of finding these different sets and carefully examining them to find a proper full set that does not eliminate important structures can, therefore, be very tedious.

Our chemical insight approach has no limitations on the structures other than linear independency. Furthermore, it does not depend on any orbital ordering. In fact, the structures’ scoring tries to follow our intuition, and the most intuitive structures would, therefore, have low scores. Thus, the chemically insightful structures are expected to all be included in the highest quality sets. Furthermore, one can select structures to be included, and the method will complement these structures to a full linearly independent set. This ensures that using the chemical insight approach, important structures will always be included, as long as they are linearly independent, with minimal effort.

### Avoiding Intra-Atomic Bonds

**Scheme 5:**
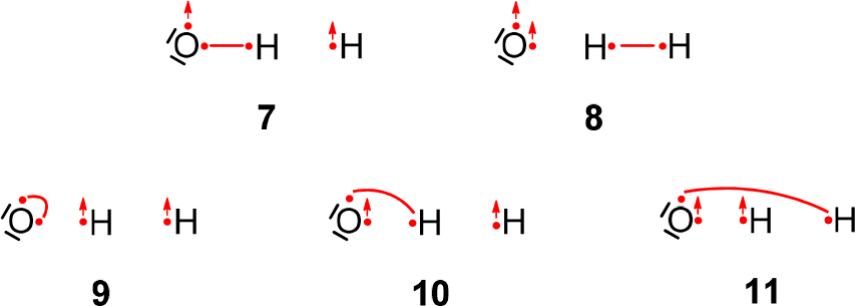
Two Most Important Structures, **7** and **8**, Required for the Reaction H + OH → O + H_2_ Using 4*e*/4o along with the Three Possible Structures That Complement Them to a Complete Covalent Set Active electrons appear as dots with lines connecting them indicating covalent bonds.

In some cases, it can be useful to avoid structures that couple spins on the same atom forming an intra-atomic bond. This is particularly true, though not limited to, for systems where one of the states involves a triplet state of that atom/fragment. The hydrogen transfer reaction, H + OH → O + H_2_, will be used to illustrate this. Three covalent structures are required to span this 4*e*/4o space, two of them, **7** and **8** in Scheme 5, are expected to be highly important, as they describe the bonds being formed or broken during the reaction. One more structure is required to span the space and complement this set. Structure **9** complements these two structures to a Rumer set. This structure however is not suitable for the description of the reaction, e.g., in its products state, as it pairs the electrons on the oxygen atom which are expected at this geometry to be unpaired in a triplet state. Structure **10** offers a better-suited structure for the description of this reaction in its different stages. This structure, however, cannot be obtained as a complementary structure for structures **7** and **8** within the Rumer formulation. In this particular case, by reordering the orbitals, it is possible to avoid structure **9** and still complete structures **7** and **8** to a Rumer set using structure **11**.

**Scheme 6:**
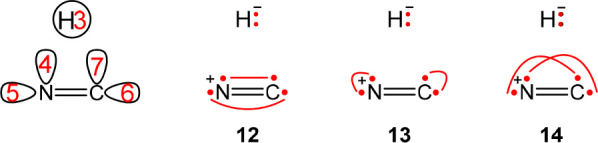
The Active Orbitals Required for the Description of Reaction HCN → CNH Using 6*e*/5o along with Three Dependent Structures That Span the Covalent Space with Two Electrons on the Hydrogen Structures **12** and **13** form a Rumer set. Active electrons appear as dots with lines connecting them indicating covalent bonds.

Table 2 summarizes the VBSCF weights obtained for the covalent wavefuction of this system in the TS geometry. All sets provide a very similar picture with around 30% and 70% contribution of structures **7** and **8**, respectively, and virtually no contribution for the third structure (being ∼1 in set 2 which is a non-Rumer set and a small-unphysical negative value (−0.3) and (−0.8) within the two Rumer sets). Furthermore, in this case a Rumer set which does not involve a structure with intra-atomic bonds could be found. This, however, is not always the case, and often Rumer sets involve several structures with intra-atomic bonds. The intra-atomic bond may also cause some anomalous behavior. To illustrate this, we will focus on the transition state of the reaction HCN → CNH using 6*e*/5o. The full so-called covalent space of this system can be divided into five subspaces accounting for the five different possible occupations of the lone pair (in the five different active orbitals, see Scheme 6). Each of these subspaces involves a set of three structures, two of which are independent. Structures **12**–**14** in Scheme 6 illustrate these structures for the subspace where the lone pair is on the hydrogen. The Rumer set that utilizes the orbital numbering given in Scheme 6 involves a structure with intra-atomic bond in each subspace.

**Table 2:** Three Possible Sets of Covalent Structures That Describe the Reaction H + OH → O + H_2_ Using the 4*e*/4o Space along with Their Corresponding VBSCF Weights (in %)

Set	ϕ_*j*_^b^	*W*_*j*_ (TS)
1^a^	7	31.5
	8	68.8
	9	–0.3
2	7	30.1
	8	68.8
	10	1.1
3^a^	7	30.0
	8	70.8
	11	–0.8

a Rumer set.

b Structures are shown in Scheme 5.

We calculated the system once using that Rumer set and once with a set of intra-atomic-free structures (i.e., structures that do not include intra-atomic bonds), obtained by the chemical insight method. The results are summarized in Table 3. It is evident that the contribution of each subspace to the overall wavefuntion, (∑*W*_*i*_), is fixed regardless of the structures that span that space. The weights of the structures with the intra-atomic bonds are often overestimated, resulting sometimes in a nonphysical negative weight for their complementary structures in the subspace. This anomalous behavior is eliminated when these intraatomic structures are excluded from the sets (Table 3).

**Table 3:** Weights (in %) of Rumer Set of Structures along with an Intra-Atomic-Free Set of Structures That Span the Covalent Space of the HCN → CNH Reaction Using 6*e*/5o Divided into Subspaces Based on the Position of the Lone-Pair

Subspace	Rumer set structures^a^	*W*_*i*_	∑*W*_*i*_	Intra-atomic-free set of structures^a^^,^^b^	*W*_*i*_	
1	[1 1 2 2] 3 3 5–6 4–7	–1.5	7.7	[1 1 2 2] 3 3 5–6 4–7	3.9	7.7
	[1 1 2 2] 3 3 4–5 6–7^c^	9.2		[1 1 2 2] 3 3 4–6 5–7	3.8	
2	[1 1 2 2] 4 4 5–6 3–7	–2.8	32.4	[1 1 2 2] 4 4 5–6 3–7	1.4	32.4
	[1 1 2 2] 4 4 3–5 6–7^c^	35.2		[1 1 2 2] 4 4 3–6 5–7	31.0	
3	[1 1 2 2] 5 5 3–7 4–6	–1.5	25.5	[1 1 2 2] 5 5 3–7 4–6	0.2	25.4
	[1 1 2 2] 5 5 3–4 6–7^c^	27.0		[1 1 2 2] 5 5 3–6 4–7	25.2	
4	[1 1 2 2] 6 6 3–4 5–7	–2.6	7.1	[1 1 2 2] 6 6 3–4 5–7	0.6	7.2
	[1 1 2 2] 6 6 3–7 4–5^c^	9.7		[1 1 2 2] 6 6 3–5 4–7	6.6	
5	[1 1 2 2] 7 7 3–4 5–6	–0.6	27.2	[1 1 2 2] 7 7 3–4 5–6	10.6	27.3
	[1 1 2 2] 7 7 3–6 4–5^c^	27.8		[1 1 2 2] 7 7 3–5 4–6	16.7	

a structures are written using the orbital occupancy of all valence electrons. Orbitals 1 and 2 present the inactive σ and π C–N bonds. Doubly occupied orbital presenting the lone pair is then given followed by the description of the two bonds. Numbering of the orbitals are based on Scheme 6.

b some of these structures can be obtained as Rumer structures using a different orbital ordering. Yet it is not possible to get all these structures together, using the Rumer rules.

c the structure includes an intra-atomic bond.

Within Rumer sets, it is possible to follow certain guidelines and reorder the orbitals (see Supporting Information) to exclude some structures with intra-atomic bonds. This process, however, is neither easy nor always possible, particularly for systems with lone pairs and radicals due to the limitations of the Rumer rules. The chemical-insight method, on the other hand, has fewer restrictions and allows therefore considerable reduction of the number of structures that include intra-atomic bonds.

### Avoiding Unphysical Negative Weights

Our last example involves the C_2_ molecule that was recently suggested to have a quadruple bonding pattern and led to a debate.^15,35−40^ 14 covalent structures are required to span this 8*e*/8o space. There are 2520 different possible Rumer sets for this system. This large number of sets suggests that there are many ways to look at this system and interpret its wave function. Therefore, when choosing a set, several considerations should be taken into account. First, one has to define the question and choose the most appropriate set. Second, the interpretation should be reliable and robust. Finally, energetic considerations should also be taken into account.

**Scheme 7:**
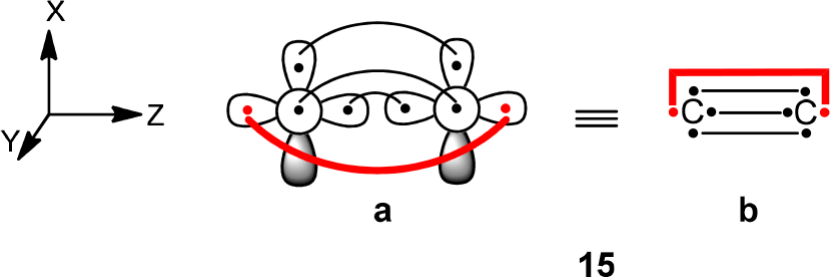
Two Different Pictorial Representations of the Quadruply Bonded Covalent Structure of C_2_ Active electrons appear as dots with lines connecting them indicating covalent bonds. (a) The orbitals in the *x* and *y*-axes are 2p atomic orbitals (AOs), while those on the *z*-axis are σ type hybrid AOs. (b) Dots above and below the C atoms represent electrons occupying the π system (p_*x*_ and p_*y*_, respectively). Dots along the *z*-axis represent σ electrons. The inverted sigma bond is the bold line.

Thus, in order to describe a quadruple bond for C_2_, the set should include structure **15** in Scheme 7. 336 out of the 2520 different possible Rumer sets of this system include structure **15**. We calculated the system using these different 336 Rumer sets. Our calculations involved localized orbitals accounting for the covalent state, as well as delocalized orbitals, accounting for both covalent and ionic contributions.

In 310 out of the 336 Rumer sets, structure **15** obtained the biggest contribution to the wave function with *W*(15) > 40%, suggesting, in agreement with Shaik et al., that the molecule has a quadruple bond.^35−37^ Yet, in all these (310) cases, some of the other structures in the sets obtained unphysical negative weights. These results were independent of whether localized or delocalized orbitals were used.

Overall, 328 out of the 336 Rumer sets, which include structure **15**, involved non-negligible unphysical negative weights that together amount to above 5% and usually (in more than 90% of the cases) above 10% while using localized orbitals. Allowing the orbitals to delocalize aggravated the situation, and 334 out of the 336 Rumer sets presented an overall unphysical negative weight that amounts to above 5% and usually (in more than 95% of the cases) above 10%. This result is problematic as negative weights, and especially such large negative weights, are meaningless and suggest that the interpretation of the wave function may not be reliable.

**Scheme 8:**
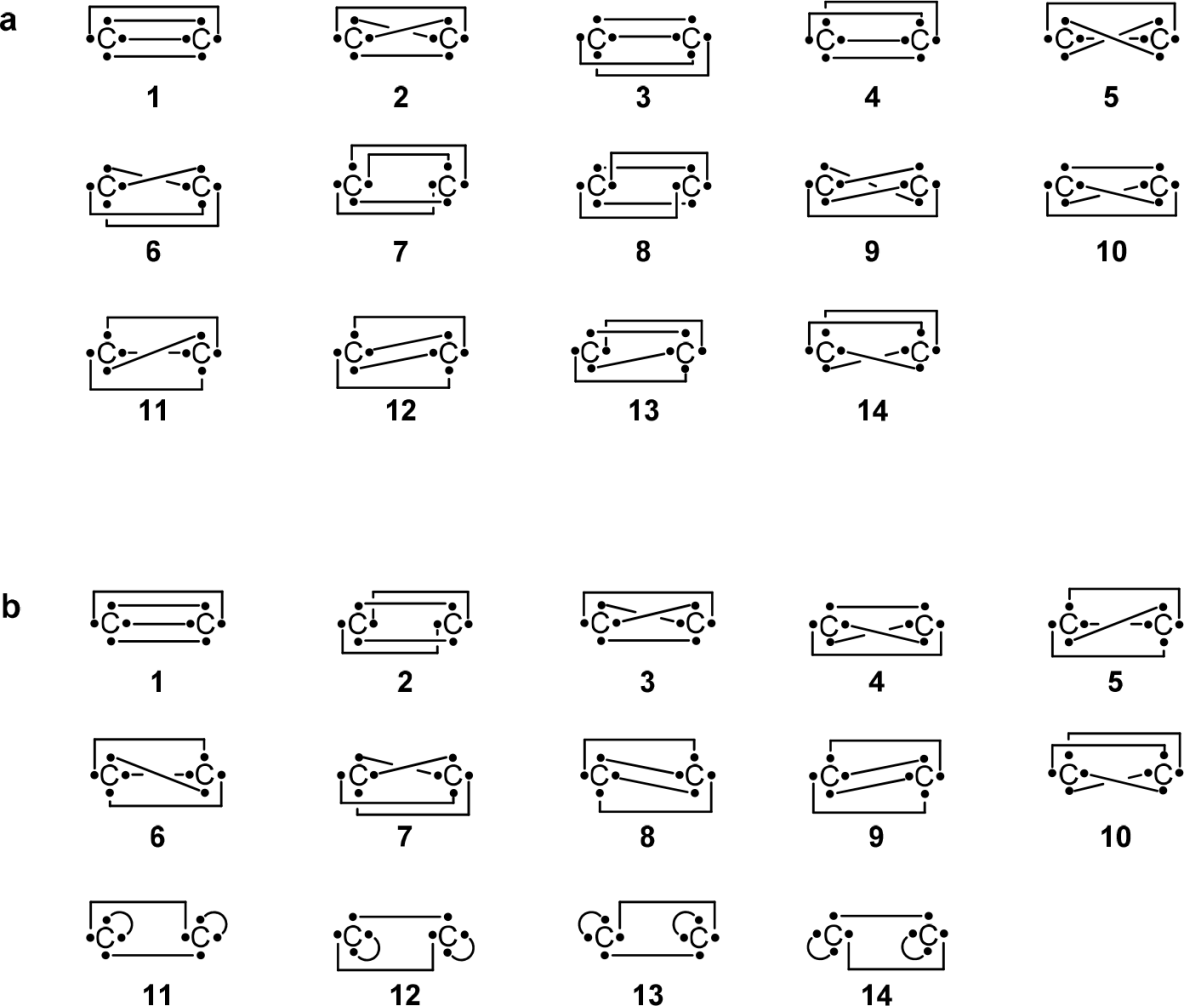
Two Representative Sets of Covalent Structures of C_2_ using 8*e*/8o: (a) Rumer Set (Table 4, 1st Entry) and (b) Chemical Insight Set which Is Not a Rumer Set (Table 4, 2nd Entry) For simplicity we follow the pictorial representation as in structure **15b** in Scheme 7.

The remaining 2 Rumer sets suggest a relatively small contribution (<20%) of structure **15** (1st entry of Table 4 and Scheme 8a), and based on their straightforward interpretation of the wave function, it is difficult to conclude that the bond is characterized as a quadruple bond. These interpretations, however, are somewhat misleading, as the potential energy surface (PES) of these sets is the same PES that served to conclude that C_2_ is characterized by a quadruple bonding based on energetic considerations.^35−37^

**Table 4:** Weight of Structure **15**, *W*(15), along with a Sum of Weights of Structures That Include the Inverted σ-Bond, ∑*W*(*σ*_inverted_), Using Localized and Delocalized Orbitals (values are given in %)

		*W*(15)		∑*W*(*σ*_inverted_)	
Set type	Entry^a^	Loc	Deloc	Loc	Deloc
Rumer	1	18	11	50	52
Chem. Insight	2	53	49	63	67
Chem. Insight	3	61	40	66	64
Chem. Insight	4	48	40	58	58
Chem. Insight	5	28	29	52	57

a Sets 1 and 2 are depicted in Scheme 8a,b, respectively. Sets 3, 4, and 5 are depicted in Scheme S3a–c, respectively, in the Supporting Information.

Finding a set that would be both reliable and compatible with the energetic considerations is important. Using the chemical insight method, the set selection for this system grows by several orders of magnitude, resulting in a vast number of different sets that include structure **15**. This enables selecting sets of structures, the interpretation of which is more reliable and balanced. The last four entries of Table 4 present four such sets (set 2 is depicted in Scheme 8b, while sets 3–5 are depicted in Scheme S3 in the Supporting Information). No significant unphysical negative weights are given to any of the structures within these sets. Furthermore, to avoid bias in the presented results, we selected sets with varying weights for structure **15**, ranging from 28−61% and 29–49%, using localized and delocalized orbitals, respectively. While this weight is not always above 40%, as suggested by most of the Rumer sets, it is still the structure with the most significant contribution in all these sets. Furthermore, summation of the weights of all structures that involve the so-called inverted σ-bond (bold bond in structure **15**, Scheme 7), increased this value to 52–66% and 57–67%, using localized and delocalized orbitals, respectively. These values are similar to a value of 67% which was reported as a possible weight for structure **15** by Dunning et al., who used the Kotani set of structures.^15,40^ However, different from the Kotani set, all the structures within our sets involve a collection of varying singlet coupled bonds and are, thus, all easily translated into pictorial chemical structures (Scheme 8b and Scheme S3 in Supporting Information).

We should stress that these results do not contradict the proposal of Shaik et al. that C_2_ has a quadruple bond but rather strengthen them. The fact that structure **15** has a non-negligible contribution suggests that it contributes to the character of the molecule. The weight depends on the identity of other structures within the sets and can, thus, change (as seen from the variety of weights within the different sets in Table 4). In general, the different sets provide us with different ways to understand and interpret the system. For some sets, the interpretation will be explicit and straightforward, while for others, it will be implicit and harder to comprehend. To reach robust conclusions, however, energetic considerations must also be taken.

The PES and the corresponding wave function are unique and common to all the different sets of structures. Thus, conclusions that are derived based on the PES are valid to that system, regardless of the set used to describe it. It is, thus, useful to use sets of structures that will explicitly give insight that agrees with the insight deduced based on energetic considerations. As shown, our approach assists in identifying such sets (Table 4, Scheme 8b and Scheme 3S in Supporting Information).

In summary, our sets suggest that there is a relatively important contribution of a quadruple bond within the C_2_ molecule, in agreement with earlier studies that utilized various energetic considerations to support it.^35−37^

## Concluding Remarks

We developed a method that constructs independent sets of spin functions with enhanced chemical insight that can be used for any VB calculation including both classical VB as well as SCGVB. The method keeps the advantage of the Rumer sets being meaningful but is much more flexible with structure choice and chemical insight that can be controlled and improved. The method imposes fewer restrictions than those imposed by Rumer rules. Hence, it overcomes Rumer sets’ weaknesses and results in sets that are better chemically adapted to the studied systems providing robust and reliable interpretations.

## Supporting Information Available

Supporting Information includes: Optimized geometries, explanations for the overall number of covalent structures and for the number of bonds in any Rumer set, Scheme of Rumer sets of C_5_H_5_ with *C*_2*v*_ symmetry, guidelines to minimize intra-atomic bonds within Rumer sets of structures as well as three non-Rumer sets of C_2_ corresponding to sets 3, 4, and 5 in Table 4 (PDF)
