# Supplementary material for: New Methodology to Produce Sets of Valence Bond Structures with Enhanced Chemical Insights
Source: J Chem Theory Comput. 2023 May 15;19(11):3102–11. doi: 10.1021/acs.jctc.2c01000 (PMC10269329; doi:10.1021/acs.jctc.2c01000)
Supplement: Supplementary file 1 — ct2c01000_si_001.pdf [file ct2c01000_si_001.pdf]

New Methodology to Produce Valence Bond Structures Sets with Enhanced Chemical Insights

Sourav Roy and Avital Shurki*

[b] Institute for drug research, School of pharmacy, Ein Kerem Campus, The Hebrew University of Jerusalem, Jerusalem, 9112001, Israel. Avital.shurki@mail.huji.ac.il

Supporting Information

Optimized geometries

C_5H_5 QCISD/6-31++g**

Atom	X	Y	Z
C	0.000000	1.192824	0.000000
C	1.172172	0.353956	0.000000
C	0.743176	-0.950655	0.000000
C	-0.743176	-0.950655	0.000000
C	-1.172172	0.353956	0.000000
H	0.000000	2.273881	0.000000
H	2.193296	0.701691	0.000000
H	1.360782	-1.836912	0.000000
H	-1.360782	-1.836912	0.000000
H	-2.193296	0.701691	0.000000

saddle point of $HCN^- \rightarrow HNC$ (QCISD/MG3)

H	-1.044148	0.255121	0.000000
C	0.080319	0.620258	0.000000
N	0.080319	-0.568095	0.000000

saddle point of $H + OH^- \rightarrow O + H_2$ (QCISD/MG3)

H	0.000000	0.000000	−0.860287
O	0.000000	0.000000	0.329024
H	0.000000	0.000000	−1.771905

C_2 (QCISD/6-31++g**)

C	0.000000	0.000000	0.695720
C	0.000000	0.000000	-0.695720

Overall number of covalent structures:

For a system of N orbitals with N electrons in a singlet state the first bond can be formed in $\binom{N}{2}$ different ways; the second bond can be formed in $\binom{N-2}{2}$ different ways and so on, until $\binom{2}{2}=1$ for the last bond. Overall, each structure contains $\frac{N}{2}$ bonds. The order of the bonds within each structure does not matter. Hence the total number of structures is divided by $\frac{N}{2}!$ which is the number of ways to arrange these $\frac{N}{2}$ bonds. Therefore, the total number of possible HLSP structures for that system is:

$$N_{HLSP} = \frac{\binom{N}{2}\binom{N-2}{2}\binom{N-4}{2}\cdots\cdots\binom{2}{2}}{\frac{n}{2}!} = \frac{\prod_{i=0}^{\frac{N}{2}-1}\binom{N-2i}{2}}{\frac{N}{2}!} \qquad \text{(S1)}$$

If the system has a nonzero spin, S, the number of unpaired electrons is $2S$, and the number of orbitals available for paring the electrons is $N-2S$. Hence, the number total number of possible HLSP structures for this system becomes:

$$N_{HLSP} = \binom{N}{2S}\frac{\prod_{i=0}^{\frac{N}{2}-S-1}\binom{N-2S-2i}{2}}{(\frac{N}{2}-S)!} \qquad \text{(S2)}$$

Here, $\binom{N}{2S}$ is the number of ways to place $2S$ unpaired electrons in N orbitals.

Finally, for a system with vacant orbitals (leading to positive charges) or lone pairs the total number of possible HLSP structures is:

$$N_{HLSP} = \binom{n}{n-N}\binom{N}{2S}\frac{\prod_{i=0}^{\frac{N}{2}-S-1}\binom{N-2S-2i}{2}}{(\frac{N}{2}-S)!} \qquad \text{(S3)}$$

where n is the overall number of orbitals, N is the number of singly occupied orbitals and $\binom{n}{n-N}$ is the number of ways to place $n-N$ lone pairs or vacant orbitals in n orbitals

Number of bonds in any Rumer set.

The number of bonds in any Rumer set, n_b^{set}, is the number of allowed bonds. In a singlet system with one electron per orbital, bonds between two odd or two even centers of the Rumer cycle (e.g. bond between centers 2 and 6) are forbidden. The reason is that an odd number of centers remains between them if such bond is formed (in the example given centers 3, 4 and 5). Therefore, at least one bond from these positions will necessarily result in crossing (with the 2-6 bond given as an

example). The one electron orbital numbering can therefore be written in a two column tableau where the first and second columns contain only the odd and even one electron orbital numbers, respectively. For a 12 electron system this results in the Tableau shown in Scheme S1.

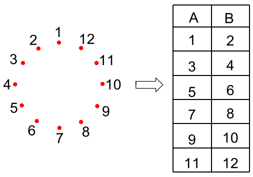

Scheme S1. Rumer cyclic scheme and the corresponding Tableau that assists in determining which bonds are permitted.

Every combination of centers between the two columns in the Tableau (one from A and one from B) forms Rumer permitted bonds. Combinations of centers within the columns, on the other hand, (i.e. both from A or B) result in forbidden bonds. Therefore, the number of permissible bonds in a singlet state with N singly occupied orbitals is $\left(\frac{N}{2}\right)^2$ which is the number of possibilities to take one number from column A and one number from column B.

Even electron systems with 2S unpaired electrons involve additional limitation, namely the distance (i.e., the numerical difference of the numbers) between the centers that form a bond cannot be larger than N-2S (a larger value leaves insufficient centers to connect with the dummy atoms – thus, necessarily leading to crossing with such a bond if formed). This accumulates to S^2 forbidden bonds. Thus, the total number of permitted bonds in systems with even number of electrons is:

$$n_b^{set} = \left(\frac{N}{2}\right)^2 - S^2 \quad \text{(S4)}$$

The number of bonds in systems with an odd number of electrons is derived similarly, leading to the following formula

$$n_b^{set} = \left(\frac{N-1}{2}\right)\left(\frac{N+1}{2}\right) - \left(S - \frac{1}{2}\right)\left(S + \frac{1}{2}\right) = \left(\frac{N}{2}\right)^2 - S^2 \quad \text{(S5)}$$

Thus, Eq. S4 presents the total number of permitted bonds, n_b^{set}, in any Rumer set for any number of electrons.

Number of allowed bonds in the chemical insight approach.

In the Chemical insight method there are no restrictions on the bonds or the bond number. The only requirement is that the set will span the space. Hence, each set can include up to $\binom{N}{2}$ bonds which is the number of ways one can choose two orbitals at a time from total N orbitals.

Rumer sets of C_5H_5 with C_{2v} symmetry

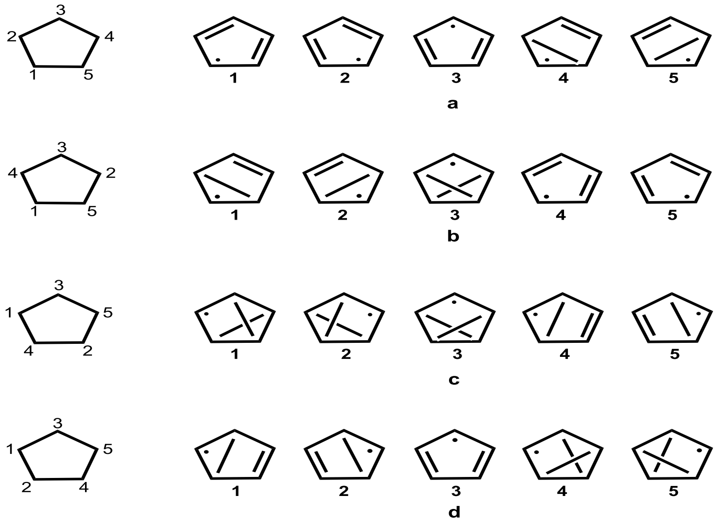

Scheme S2. The four possible C_{2V} symmetric Rumer sets along with the orbital numbering. Set S2a is identical to set 2e in the main text.

As can be seen all symmetric sets with the exception of set S2a involve crossing of bonds, and are thus, less intuitive.

Minimizing intra-atomic bonds in Rumer set of structures

Intra-atomic bonds can be avoided within Rumer sets only for systems with one electron per orbital. For all other systems (systems with active lone pairs) the number of structures with intra-atomic bonds can only be reduced but cannot be completely eliminated. Following are guidelines to minimize intra-atomic bonds within Rumer set of structures:

- In simple systems, with no lone pairs (1e per orbital), bonds are generated between odd and even numbered orbitals. Therefore, to avoid intra-atomic bonding the numbers of orbitals on the same atom should either be both odd or both even.

- In case of radicals, numbering the two orbitals on the same atom using the first and last numbers (not including the dummy) will avoid any bonding between these two orbitals.
- When lone pairs are included, avoiding all intra-atomic bonding is not possible. The minimum number of intra-atomic bonding will be obtained when orbitals on the same atom will be assigned the numbers n, n+2 (in this case only when the lone pair will be on orbital n+1 an intra-atomic bonding will appear).

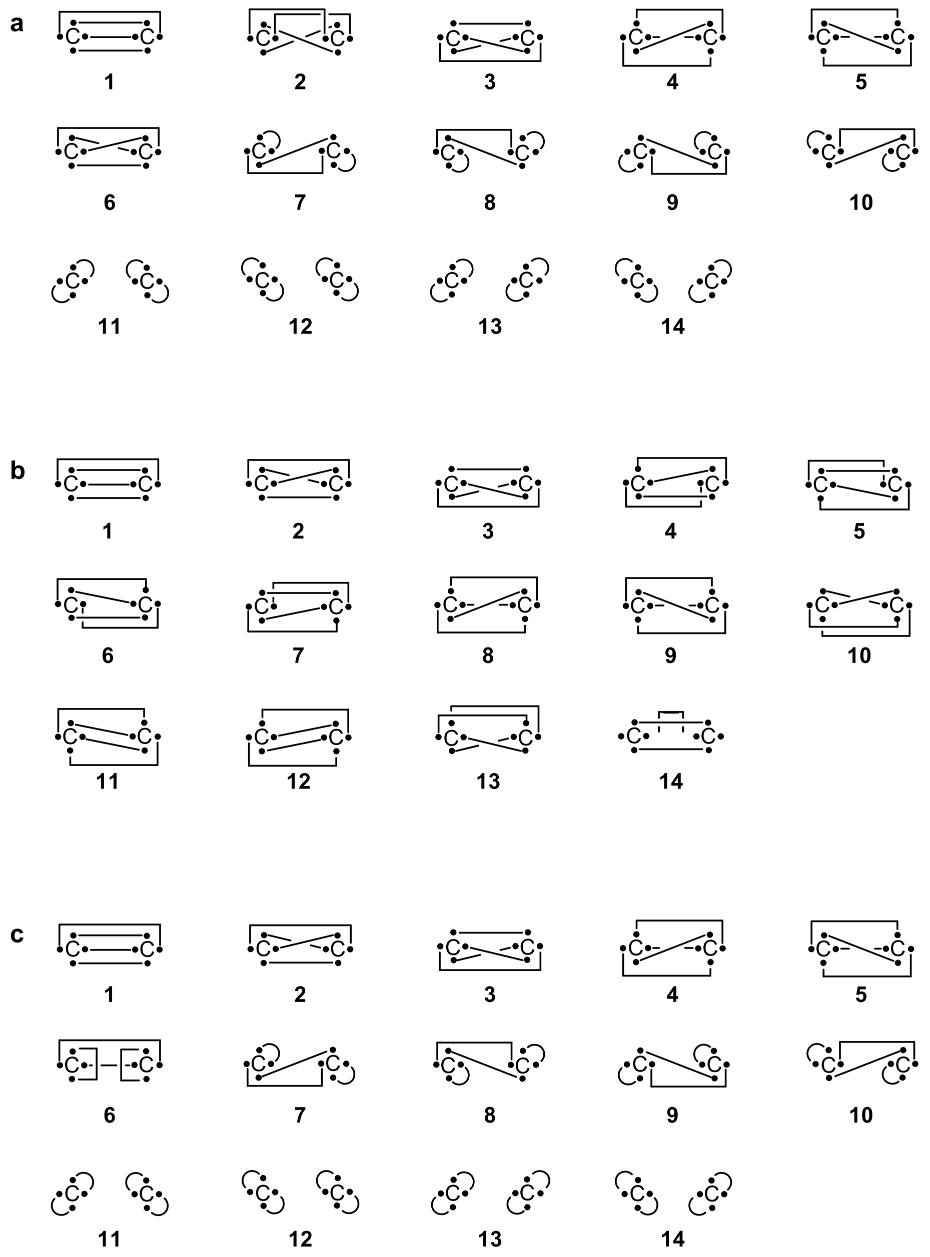

Scheme S3. Three non-Rumer sets of C_2 corresponding to sets **a**. 3 **b**. 4 and **c**. 5 in Table 4 of the main text. For simplicity, we follow the pictorial representation as in structure **15b** of Scheme 7 within the main text.
